# Challenges in the Diagnosis and Management of Fibrotic Hypersensitivity Pneumonitis: A Practical Review of Current Approaches

**DOI:** 10.3390/jcm11061473

**Published:** 2022-03-08

**Authors:** Teng Moua, Tananchai Petnak, Antonios Charokopos, Misbah Baqir, Jay H. Ryu

**Affiliations:** Division of Pulmonary and Critical Care Medicine, Mayo Clinic, Rochester, MN 55905, USA; petnak@yahoo.com (T.P.); charokopos.antonios@mayo.edu (A.C.); baqir.misbah@mayo.edu (M.B.); ryu.jay@mayo.edu (J.H.R.)

**Keywords:** hypersensitivity pneumonitis, lung fibrosis, interstitial lung disease

## Abstract

Recent advances in fibrotic hypersensitivity pneumonitis include improved diagnostic guidance, systematic assessments of immunosuppressive therapy, and the recent availability of antifibrotic therapy (nintedanib) for those with progressive disease. A standardized approach to diagnosis may lead to better inclusion criteria for future therapeutic protocols and delineation of disease or treatment response predictors for real-world management. This review will highlight current diagnostic and treatment challenges and remaining knowledge gaps or areas of uncertainty, with a practical overview of supporting evidence and its clinical implications. Exposure history, serologic testing for antigen sensitivity, bronchoalveolar lavage lymphocytosis, histopathology, and radiologic findings will be covered in the diagnosis section, with immunosuppression, antifibrotic therapy, lung transplantation, and disease prognosis in the treatment and management section.

## 1. Introduction

Hypersensitivity pneumonitis (HP) is characterized by sensitization to inhaled environmental organic and inorganic antigens measuring approximately 5–10 microns that are able to deposit in the alveoli or small airways and cause immune-mediated lung injury [1,2,3,4]. A result of recurrent and sometimes occult exposure to inciting environmental antigens, associated morbidity and mortality in those with advanced fibrosis may be comparable with idiopathic pulmonary fibrosis (IPF) [5] or other fibrotic interstitial lung diseases (f-ILDs) [6,7,8]. Prior disease categorizations described ‘acute’, ‘subacute’, or ‘chronic’ HP presentations [9], though what specific disease components were being considered for such designations was unclear [10]. For example, some patients with ‘chronic’ HP may not have evidence of radiologic fibrosis, while some classified as ‘subacute’ may have early traction bronchiectasis or reticular changes, now considered fibrotic in terms of classification. Recent international diagnostic guidelines now categorize HP into fibrotic vs. non-fibrotic forms based on imaging or histopathologic assessment [11,12]. Our review will use the new classification schema of fibrotic vs. non-fibrotic, rather than ‘acute’ vs. ‘chronic’ given the nuances above. In this review, we will focus specifically on fibrotic HP (f-HP) noting that diagnostic and treatment principles described below may also be applicable to non-fibrotic HP.

Advances have been made in recent years regarding diagnostic evaluation and management strategies in patients with suspected f-HP, with many clinical challenges still remaining. These include identification of inciting antigen(s), the diagnostic relevance of serum precipitin or antigen-specific antibody testing and bronchoalveolar lavage (BAL) lymphocytosis, overlap of histopathologic and radiologic features with other f-ILDs, and therapeutic decision-making in the absence of controlled treatment studies. Even with recently established international consensus guidelines [11,12], diagnostic confidence in individual patients may vary based on clinician judgment or multidisciplinary team discussion (MDD). Current treatment strategies include antigen avoidance when the inciting agent is identified and chronic immunosuppression and/or antifibrotic therapy based on the extent of fibrotic findings or rate of progression. Controlled studies are lacking regarding the short or long-term therapeutic efficacy of these approaches. This review encapsulates recent advances and remaining challenges in the diagnosis and management of f-HP as encountered in clinical practice.

## 2. Diagnosis: Where the Whole Is Greater Than the Sum of Its Parts

### 2.1. Exposure History

Several factors are involved in the assessment of exposure history. These include clinician awareness and suspicion for relevant antigens, patient recall of potential exposures, and temporal association with presenting clinico-radiologic findings. In particular, temporal association between exposure and lung disease may be more difficult to identify in patients with f-HP due to the chronic or recurrent nature of exposures. A recent survey of US ILD specialists highlighted additional barriers to antigen identification using nominal group technique. These barriers included uncertainty with the clinical significance of reported or solicited exposures, inability to test or objectively confirm historical associations, and occult or unsolicited exposures which may not allow for confident exclusion of HP [13]. It is also unclear whether antigen subtype (avian vs. mold, for example), exposure intensity, or duration of exposure contribute to greater fibrosis.

Cases of acute or subacute HP (non-fibrotic) often rely on temporally related exposure and symptoms for diagnosis, with clinical improvement after exposure abstention and recurrence with re-exposure increasing diagnostic confidence. Use of the ‘environmental challenge’ where patients report improvement or abatement of symptoms when away from culprit settings or source objects and worsening after returning have been reported in f-HP. The temporal evolution of exposure-related symptoms though may take days or weeks to recur rather than several hours or only a few days [14]. One study suggested a two-week abstention with close monitoring of several clinical and laboratory findings may support a diagnosis of f-HP compared to non-HP controls. The authors described measured improvements in forced vital capacity (FVC), C-reactive protein, and body temperature as specific parameters [15].

Correlation of exposure intensity or extent with serologic results or disease course have been previously reported. In a study of 19 patients (15 of which were fibrotic) with HP, mold samples were taken from home and work environments with species-specific comparison to standardized serologic testing. Positive serologic testing was found in 12 with only one patient having matching or overlapping antigen burden found in the environment. Using clinical interviews or solicitation for a potential antigen, environmental assays were also more likely to be positive in those with suspected disease than in control subjects. Correlation of standard panels with identified antigens from environmental testing was not found in the majority [16]. Quantified environmental avian antigen appeared to correlate with disease progression and outcome over one year in another study involving 23 subjects with bird-related HP [17]. Higher levels of indoor avian antigen were also detected in the homes of those with bird-related HP and asymptomatic breeders compared to healthy controls. Diverging clinical manifestations were noted despite similar intensity of exposure in those with diagnosed disease and asymptomatic breeders [18]. Avian antigen appears to be the most commonly implicated antigen type [19,20] with the greatest number of reported cases or cohort studies to date in the published literature [21,22]. Some studies have suggested better survival in those with avian-related HP compared to mold or other antigen subtypes [23].

To better unify an approach to exposure solicitation in f-HP, a multinational Delphi consensus statement was recently formulated highlighting ≥90% agreement on 18 points of historical questioning, ranging from the presence of a ‘moldy smell’ in the environment to water damage in the home or ownership of feather-containing items [24]. The individual frequency and specificity of these findings in clinical practice will vary according to local population and geographic characteristics. While we support the use of evidence or consensus-based questionnaires for soliciting potential exposures, such findings on their own without additional histopathologic or radiologic evidence may not on its own lead to higher diagnostic confidence. A standardized approach though may lead to improved antigen identification and subsequent avoidance, an important starting point for both diagnosis and management.

### 2.2. Serum Precipitin Antibody Testing

Serum precipitin antibody testing is often obtained in f-HP to identify prior exposure and sensitization to potential inciting antigens [25,26]. Such testing may be comprised of several mold, bacterial, or avian antigens, on one panel, with varied standardization between laboratories or order sets. Sensitivity and specificity for individual tests will also vary based on the study-specific criteria for HP diagnosis [27,28]. Until recently, the diagnosis of HP including those with fibrosis varied according to individual study criteria and contributed to a wide range of intrinsic positive and negative test characteristics for determining sensitivity and specificity. Variation in standardization or positive cut-offs does not appear to be a leading factor in whether clinicians obtain serologic testing but rather their positive or negative predictive characteristics in the context of disease prevalence and pre-test suspicion [29]. When background disease prevalence or pre-test suspicion is low, positive findings may be considered either evidence of prior antigen sensitization but clinically irrelevant, or spuriously positive and ignored.

It remains unclear whether serum precipitin levels are higher during active antigen exposure with acute disease compared to lower or waning titers with antigen avoidance or directed treatment [29,30]. In one study assessing serum IgG or IgA to several bird-related proteins, measured serum precipitin levels were notably higher in those with newly diagnosed acute disease and those with recurrent flares or progressive clinical symptoms compared to controls [31]. The combination of several avian antigen species in this study also improved the sensitivity and specificity of serologic testing for HP diagnosis. In a large study of 647 patients with suspected HP (fibrotic and nonfibrotic), positive IgG levels above the reference value increased the likelihood of subsequent HP diagnosis nearly 10-fold [32]. False-positive rates as reported in the sera of 10,000 patients undergoing screening was approximately 2%. Of those with positive findings, 58% were clinically diagnosed with HP [33]. An ‘a la carte’ approach to serologic testing based on institutional or regional prevalence of inciting antigens may be a reasonable approach to minimize potential false positives.

Given the variation in commercially available tests among community practices and referral centers, we support the use of serum precipitin antibody testing as either a screening test to support prior antigen sensitization in those with undifferentiated ILD, or to potentially delineate culprit antigens in those with otherwise diagnosed disease. A positive avian panel, for example, might prompt a more in-depth evaluation for historic or active exposure to bird protein, including down or feather-containing items in the home or bird droppings in the work environment. This may not have been previously pursued in the absence of clinical suspicion without positive testing. A positive serologic result on its own again would be insufficient for confident HP diagnosis, nor would a negative test necessarily exclude disease. Evidence of antigen sensitization will need to be assessed with elements of exposure history, disease tempo or course, radiologic findings, histopathology when available, and adequate exclusion of other diagnostic contenders.

### 2.3. Bronchoalveolar Lavage Lymphocytosis

The concept of elevated lymphocyte count in the BAL of patients with HP dates back several decades with initial studies noting higher lymphocyte count compared to other diffuse lung diseases [34,35,36,37,38]. This finding was first observed in what appeared to be acute or subacute presentations of HP, with systematic exploration occurring only recently in those with fibrotic disease [39]. Prior studies also described transition from initial neutrophilic to lymphocytic BAL predominance, as driven by both type III and type IV hypersensitivity immune responses [35,40,41]. BAL neutrophilia was recently reported in a cohort of patients with bird-related chronic or f-HP after directed inhalational challenge [42,43].

Attempts at systematically assessing the degree and type of alveolitis in those with f-HP may be confounded by incorporation bias, where BAL lymphocytosis was often a diagnostic criterion for enrollment into studies assessing this and other unrelated issues. Additional confounding may also involve the unclear differentiation of fibrotic vs. non-fibrotic disease with the use of prior classification schemes (acute, subacute, and chronic), where BAL lymphocytosis findings may intrinsically vary.

Recent systematic reviews and meta-analyses have pooled the extent of BAL lymphocytosis in patients with non-fibrotic and fibrotic ILDs and found higher lymphocyte percentages in f-HP compared to IPF and sarcoidosis [44]. Increased positive cut-offs lead to better specificity but lower sensitivity for the diagnosis of HP. Sensitivity and specificity was 69% and 61%, respectively, for the more clinically challenging distinction of f-HP from IPF using BAL lymphocyte percentage [44]. Similar sensitivity and specificity were described in a meta-analysis of fibrotic or previously ‘chronic HP’ using a BAL lymphocyte percentage cut-off of 20%, noting older age and prior smoking was associated with lower BAL lymphocyte percentages [45].

A recent Delphi statement proposed a BAL lymphocyte cut-off of greater than 40% as supportive of f-HP (“chronic”) diagnosis [46] while the 2020 ATS/JRS/ALAT diagnostic guideline supported a cut-off of greater than 30% within the context of other clinical and radiologic findings [11]. The 2021 ACCP guideline suggested pursuing BAL lymphocyte assessment in specific scenarios where a positive finding may help clinicians reach particular diagnostic thresholds [12]. Lastly, a recent study suggested detection of fungal DNA was greater in the BAL fluid of those with home-related HP compared to healthy controls or other non-HP lung diseases and might further support HP diagnosis [47].

BAL lymphocyte percentage in f-HP may have also have a prognostic role where higher levels predict better survival suggesting more inflammatory or less fibrotic disease [48]. Prior studies suggest an association between radiologic fibrosis and BAL findings, with lower BAL lymphocyte percentage being associated with increased radiologic honeycombing [48,49]. While of interest, clinically symptomatic or worsening patients (in the absence of infection) will likely be offered directed immunosuppressive and/or antifibrotic treatment without deference to BAL findings. Further studies are needed to review the background frequency or incidence of elevated BAL lymphocytosis in f-HP, noting recent evidence suggests a lower frequency in those diagnosed per guideline-based criteria without incorporation bias [50].

### 2.4. Computed Tomography (CT)

As exposure history is often narrative and dependent on clinician suspicion or patient recall, and both BAL and serologic findings have varied sensitivity and specificity, radiologic findings may be more objective in the diagnosis of patients with suspected f-HP. This is highlighted by the 2020 ATS/JRS/ALAT diagnostic algorithm that places radiologic findings before BAL lymphocytosis and histopathology. Highly cited characteristic high-resolution CT findings for HP include centrilobular nodularity, mosaic attenuation and the “headcheese” or “three-density” sign [51,52], with peri-airway or bronchovascular distribution of reticulation and upper or mid-lung distribution. Although such findings may be more characteristic of HP [53] than other ILDs they are still found variably in diagnosed cases of HP. Lynch and colleagues reported 3 of 27 patients (11%) with ’chronic’ HP had typical usual interstitial pneumonia (UIP) pattern on CT, indistinguishable from IPF [54]. Interestingly, one series reported radiologic emphysema in HP patients who were non or ex-smokers, with varying radiologic severity, upper lobe predominance, and emphysema morphology ranging from centrilobular to bullae [55].

A more commonly recognized CT feature favoring f-HP versus IPF is air trapping or mosaic attenuation. However, up to 50% of patients with IPF may also manifest air trapping particularly in more fibrotic areas of the lung, while the “headcheese” or “three-density” sign was more specific (0.93) but less sensitive for f-HP (0.49) [51]. Airway-centric fibrosis seen in both the upper and lower lobes was found in all biopsy-confirmed cases of f-HP in one series, suggesting it may be an important element in radiologically differentiating f-HP from other f-ILD [56]. Other imaging characteristics favoring f-HP include upper and mid-lung predominance, along with superimposed ground-glass attenuation and diffuse nodularity [57]. Disease progression in HP is variable. Fibrotic progression was reported in 86% of patients over a median follow-up of 4.9 years in a retrospective analysis of 91 patients with ’chronic’ classification [58].

Radiologic findings also have prognostic significance [59,60], particularly honeycombing and mosaic attenuation or air-trapping [61,62]. The latter was associated with better survival in one series [52], while f-HP with radiologic honeycombing had similar survival to IPF patients with honeycombing (2.76 vs. 2.81 years from time of diagnosis), as compared with non-fibrotic HP (>14.7 years) or f-HP without honeycombing (7.9 years) [61]. Correlation of radiologic and histopathologic findings in terms of disease activity has been described [63]. More radiologic fibrosis has been shown to correlate with lower BAL lymphocyte percentage [48], though unclear in terms of predicting outcome given their inverse correlation.

In summary, we suggest radiologic findings and distribution patterns associated with f-HP may be more objective and characteristic than patient-reported exposure history or positive serology, noting overlap may be seen with other f-ILD (honeycombing and traction bronchiectasis, for example). Sensitivity for more specific findings may also be low (mosaic attenuation or head-cheese sign) in terms of supporting HP diagnosis. A strongly suggestive radiologic pattern combined with positive exposure history increases diagnostic confidence from ‘low’ to ‘moderate’, and to ‘high’ with the addition of BAL lymphocytosis, according to recent diagnostic guidance [11].

### 2.5. Histopathology

Characteristic histopathologic findings for f-HP include centrilobular or airway fibrosis, bridging fibrosis, poorly formed granulomas in both the airway and interstitium, and increased interstitial cellularity, as highlighted recently by international consensus [11]. However, the spectrum of histologic findings varies widely in individual cases [64,65], and no specific histopathologic finding denotes duration of disease beyond perhaps more UIP-like features correlating with greater chronicity. One study suggested 17.6% of those with short-term clinical disease duration (<6 months) already had early fibrotic changes on biopsy [66,67]. Diagnostic confidence was ranked in that study based on the presence of specific histopathologic findings, which were dominated by cellular bronchiolitis with interstitial pneumonia, interstitial granuloma, and air space multinucleated giant cells in the majority [66].

Various interstitial pneumonia patterns have been described in patients with f-HP including UIP, nonspecific interstitial pneumonia (NSIP), and organizing pneumonia with fibrosis. Centrilobular and bridging fibrosis may distinguish UIP associated with f-HP from that of IPF [68], with giant cell or granulomatous findings more suggestive of subacute or active inflammatory disease [64], found equally in the airspace and interstitium [69]. One study of f-HP patients with UIP-like histopathology suggested the extent of fibroblast foci correlated with extent of pulmonary function abnormality and radiologic fibrosis, as similarly reported in IPF [70], and may pose as histologic predictors of outcome [63]. Another study suggested similar survival between those with UIP and NSIP-like histologic patterns (2.8 vs. 2.1 years), with those having only peribronchiolar fibrosis reporting a better median survival of 11.3 years [71].

Kappa agreement between expert pathologists regarding which features in combination or individually suffice for a confident diagnosis of HP has historically been low. In this setting, additional radiologic and clinical findings may support the diagnosis of HP more than histopathologic findings alone, though current diagnostic guidelines value the distinctiveness of consistent histopathologic findings given the difficulty of obtaining relevant exposure history or overlapping radiologic findings in many patients. One study suggested about a third of f-ILD cases with both clinicoradiologic and histopathologic findings remained unclassified after MDD, with older age and radiologic and histologic findings typical of UIP correlating with IPF diagnosis compared to histopathologic findings of increased peribronchiolar metaplasia and giant cell/granulomas suggesting f-HP [72]. Those with histopathologic features suggestive of HP may also be rediagnosed after additional MDD review as other non-HP ILDs, often due to discrepant radiologic or BAL findings [73]. Patients with suggestive exposure history or positive serology but atypical radiologic findings may require histopathology to confidently diagnose HP, introducing attendant procedural risks and morbidity [74]. The value of typical or supportive histopathology is highlighted in the recent consensus guideline, suggesting ‘high’ or ‘definite’ diagnostic confidence even in the absence of typical radiologic findings or exposure history [11]. Indeed, we support obtaining histopathology, if consistent with a patient’s wishes and of tolerable risk, where clinical and radiologic findings are indeterminate or incomplete and higher diagnostic confidence cannot be achieved without it.

### 2.6. Current Consensus Diagnostic Guidance

Prior diagnostic classification of HP involved categorization of presentations or ‘phenotypes’ into ‘acute’, ‘subacute’, or ‘chronic’, though specific designation of which clinical component this was referring to (symptom duration, exposure duration, or extent of radiologic findings?*)* was unclear [9,10,75]. Inferences based on radiologic findings were often used, combining both non-fibrotic and early fibrotic findings such as mosaicism or the “headcheese” sign into those considered ‘subacute’ [9], and those with more advanced fibrotic changes with fibrotic NSIP or UIP-like radiologic patterns as ’chronic’. This historical classification again combined patients with varied presentations across a broad spectrum of disease, making meta-analyses or pooling of associated data difficult to interpret.

A first attempt at standardizing diagnostic criteria for f-HP (“chronic”) was presented by Morisset and colleagues in 2018 as an international Delphi statement [46]. Applying levels of diagnostic ontology as confidence levels [76], the approach involved starting with clinical suspicion based on exposure history followed by hierarchical radiologic, BAL, and biopsy findings. A similar approach of building diagnostic confidence based on the availability and consistency of individual findings was also used for the 2020 ATS/JRS/ALAT [11] and 2021 ACCP [12] clinical practice guidelines. While the ATS/JRS/ALAT guideline appeared to suggest higher diagnostic confidence with histopathology, the ACCP guideline gives greater weight to exposure history and radiologic findings, where consistent findings for both may preclude invasive studies such as bronchoscopy or lung biopsy. A unifying element of MDD is still advised, though f-HP had poor MDD agreement compared to other f-ILD prior to current consensus guidance [77,78].

Distribution of diagnostic confidence levels for f-HP in practice will likely vary given the spectrum of individual clinical findings and the availability of more invasive diagnostic findings such as BAL or biopsy where there is equipoise or overlap with other ILDs. One series using an older diagnostic algorithm found only 9.9% of all biopsy-proven f-HP cases met consistent or ‘definite’ diagnostic confidence levels for f-HP based on elements of exposure history, serologic results, HRCT findings, and BAL analysis; a majority of cases met only ‘probable’ or ‘possible’ HP criteria [79]. Guler et al. applied an adapted version of the algorithmic approach proposed by Morisset and colleagues [46] and found good specificity (0.90) and sensitivity (0.74) when an acceptable diagnostic confidence level of ≥70% was used, but for ‘definite’ diagnosis (confidence >90%), sensitivity was much lower (0.35). This may be the result of varied interpretation of individual diagnostic elements or their non-availability for meeting higher confidence thresholds in clinical practice [80]. Only 50% of MDD-diagnosed cases in another study of 251 initially undifferentiated ILD had diagnostic confidence >50% for f-HP using a similar algorithmic approach, mostly due to the absence or incompleteness of clinical or radiologic findings despite all patients having undergone histopathologic confirmation [81]. Barber et al. reported a survey of British clinicians who described only 32% of their suspected HP cases had identifiable causative antigens, with 40% undergoing BAL and 10% undergoing surgical biopsy [82].

Varied use and clinical value attributed to certain diagnostic elements may bias decision-making in both algorithmic and MDD approaches [82,83]. For example, a plausible exposure history (pet bird owner) and typical radiologic findings may lead to a confident diagnosis without additional invasive testing, while initially unclassifiable ILD may still have a broad differential diagnosis that only tentatively includes f-HP if exposure history is dubious, a UIP-like CT pattern is encountered, and histopathology is only suggestive but not definitive for HP.

## 3. Treatment, Management, and Prognosis

### 3.1. Antigen Avoidance

Antigen avoidance in managing patients with f-HP is often confounded by the inability to identify culprit antigen(s) or exposure setting(s). Confirmatory testing such as inhalational challenge [84,85] may support the identification of specific antigens but are not standardized or widely available. Professional inspection and testing of home or work environments may provide data on the presence of potential mold or bacterial species, though specific testing for avian or other organic proteins appears limited to research efforts [18]. Prior studies have reported on the clinical effects of antigen identification and avoidance with varied results. De Sadeleer and colleagues found no difference in outcome among patients with f-HP who avoided antigen compared to those with unidentified antigen or unreported avoidance [86]. In contrast, Fernandez-Perez and colleagues reported antigen avoidance in those with ‘chronic’ (fibrotic) HP having improved outcome compared to those whose antigen or source remained unidentified [87]. Other studies suggest a mixed response to antigen avoidance [88], perhaps with confounding by early vs. later fibrotic stage disease and the effects of directed treatment.

A recent scoping review of the literature involving 205 cases of HP suggested patients used various maneuvers to avoid antigen exposures. These included removing potential source items from the environment, quitting occupations with potential exposures, moving out of suspected homes, abstaining from suspected hobbies or recreational settings, and pursuing abatement protocols to thoroughly clean or remove potential antigens [89]. Additional studies are needed to assess the effect of antigen abatement or avoidance on more immediate signs of clinical disease, including short-term measures of lung function, radiologic findings, and symptoms.

### 3.2. Immunosuppressive Therapy

Only one controlled clinical study completed decades ago in patients with what may be classified as ‘acute’ to ‘subacute’ HP (Farmer’s lung) found stability in FVC and improvement in diffusing capacity of lung for carbon monoxide (DLCO) with corticosteroid treatment compared to placebo. Its effects appeared to wane by one year with increased rate of recurrence observed in those initially treated [90]. No additional controlled studies have since been performed to assess the role of corticosteroids or steroid sparing agents (SSA) in improving or halting disease progression.

In recent years, several large single and multicenter retrospective assessments of treatment with corticosteroid and SSAs such as mycophenolate and azathioprine have been pursued noting overall stabilization of FVC with varied improvement in DLCO. Morisset and colleagues reported a single center experience involving 70 patients treated with either mycophenolate or azathioprine over a mean follow-up of 11 months, confirming stabilization of FVC with upward trend in DLCO [91]. Adegunsoye et al. compared the PFT trajectory of patients treated with SSA at several institutions, noting those who received SSA had worse long-term outcomes while FVC and DLCO trended towards stability after corticosteroid initiation [92]. Both studies used prior ‘chronic’ classification nomenclature with only one providing a descriptor of the extent of radiologic fibrosis, including honeycombing and CT fibrosis scores [92]. One series suggested improvement over two years in FVC and total lung capacity but no change in DLCO with azathioprine [93]. Another single-center study highlighted the potential benefit of slowing disease progression with corticosteroids, though treatment duration and dosing varied despite the use of a propensity matched untreated cohort [94]. Adjustments for baseline characteristics such as age, smoking history, antigen avoidance, or lung function at the time of treatment initiation have not been systematically assessed. One retrospective study found higher BAL lymphocytosis to be predictive of treatment response [48].

Equipoise remains regarding the utility of response to an initial corticosteroid burst as a marker of response or stability with long-term SSA treatment. It also remains unclear whether long-term use of SSAs improve outcome or survival more in certain patients compared to others, perhaps those with less fibrosis and more mosaicism suggesting active inflammation or ongoing antigen exposure. Morbidity associated with immunosuppression in IPF has been found to be associated with telomere length or short telomere syndrome [95,96]. Telomere-related phenomena has also been described in f-HP [97], with a recent multicenter cohort study suggesting f-HP patients with short telomeres had worse survival and less response to mycophenolate [98]. Other immunosuppressant agents or biologics reported in f-HP include rituximab [99,100] and leflunomide [101]. A multicenter study reported the use of rituximab in a cohort of 20 patients with ‘chronic’ HP, though selection criteria appeared broad and included those with less severe disease as defined by more mild FVC abnormality. Rituximab therapy was well-tolerated and stabilization to mild improvement of lung function was observed.

With consensus diagnostic guidance now available to establish clear inclusion and exclusion criteria, large controlled multicenter studies performed over several years (necessary to power comparative endpoints due to the slower progression of disease in many patients) may be feasible and are needed to determine the true effect of chronic immunosuppression. Until then, current lower quality evidence reflects the historical use of initial corticosteroids followed by more long-term SSA in patients with progressive disease. Indeed, we highlight close follow-up to determine therapeutic response and monitoring of any adverse effects, with modification or discontinuation of ineffective or poorly tolerated treatment as indicated.

### 3.3. Antifibrotic Therapy

The utility of antifibrotic therapy in f-HP was demonstrated in a large multicenter controlled study involving nintedanib and all forms of non-IPF progressive f-ILD, defined as progressive prior to study enrollment based on a combination of clinical, radiologic, or pulmonary function parameters [102]. Patients with f-HP comprised the largest subgroup of this study and experienced less decline in FVC with the use of nintedanib compared to placebo [103]. Based on these findings, nintedanib has been approved for f-HP with a progressive phenotype, with greater benefit in terms of slowing FVC decline in those with more UIP-like radiologic or histopathologic findings. Prior treatments such as corticosteroids and SSA were not excluded during trial initiation, noting nintedanib appeared to have an independent effect on FVC decline. A proof-of-concept open-label study involving 13 patients with f-HP treated with pirfenidone for one year reported stability in FVC with minimal adverse effects compared to control [104]. For both, effect on mortality remains uncertain and has not been systematically assessed.

### 3.4. Transplantation

Although lung transplantation has been reported in patients with progressive f-HP, IPF still accounts for the majority of ILD indications. This may be reflective of perhaps more stable disease or the slower progression of f-HP. Transplant diagnoses have been occasionally attributed to IPF with f-HP found unexpectedly after transplantation on histopathologic examination of the explanted lung, in one series up to 16% [105]. The post-transplant clinical course of those with f-HP appears similar to those with other f-ILDs and better than those with IPF [105], with limited reports of disease recurrence after transplantation [106].

### 3.5. Prognosis

Several large cohort studies report the long-term prognosis of f-HP (again previously described as ‘chronic’) [107,108,109], highlighting clinical predictors of age, FVC, and radiologic honeycombing. Such findings are reflected in one study applying the Gender-Age-Physiology (GAP) Index, a prognostic model originally derived in patients with IPF and applied to other non-IPF chronic interstitial lung disease patients including 206 patients with ‘chronic’ HP, noting similar staging and predictive characteristics [110]. Another study found a median survival of seven years highlighting older age, lower BAL lymphocyte counts, and DLCO as predictors of poorer outcome [107]. Alberti and colleagues compared the relative survival of f-HP and IPF in their retrospective single center study and found similar Kaplan-Meier estimators of two-year survival after adjusting for age and FVC at the time of diagnosis [108]. By comparison, prognosis in those with less fibrotic disease appears better than IPF, considering lead time bias may be relevant [52].

Depending on extent of fibrosis at the time of diagnosis, there is evolving evidence that those with UIP-like findings or honeycombing have similar outcomes to IPF [59]. Only a few studies have differentiated survival outcomes based on histologic subtype [107], with those having UIP-like findings and less cellularity and granuloma formation having poorer outcomes [71]. Lastly, acute exacerbation characterized by punctuated decline over several weeks with worsening clinical and radiologic findings has been reported in f-HP [111], occurring in up 18% over a median follow-up of 30 months with an in-hospital mortality of 44% in one series [112].

## 4. Summary

Hypersensitivity pneumonitis has been recently classified as fibrotic vs. non-fibrotic based on radiologic or histopathologic findings. Given the chronicity of disease and therefore unclear temporal association with culprit antigens, f-HP continues to pose diagnostic and management challenges. Recent diagnostic advances include better characterization and standardization of algorithmic approaches using aggregate findings that meet diagnostic confidence levels, which may improve disease definition or inclusion criteria for both prospective and retrospective studies involving f-HP. Unfortunately, ongoing challenges include overlapping or difficult to verify historical or narrative elements, low-level evidence for current immunosuppressive treatment strategies, and concerning long-term outcomes that may be as morbid or severe as other progressive fibrotic lung diseases.

## Data Availability

Not applicable.

## Abbreviations

ACCP	American College of Chest Physicians
ATS/JRS/ALAT	American Thoracic Society/Japanese Respiratory Society/Asociación Latinoamericana del Tórax
BAL	bronchoalveolar lavage
DLCO	diffusion capacity of lung for carbon monoxide
f-HP	fibrotic hypersensitivity pneumonitis
f-ILD	fibrotic interstitial lung disease
FEV1	forced expiratory volume in the first second
FVC	forced vital capacity
HP	hypersensitivity pneumonitis
ILD	interstitial lung disease
IPF	idiopathic pulmonary fibrosis
MDD	multidisciplinary team discussion
NSIP	nonspecific interstitial pneumonia
SSA	steroid sparing agent
TLC	total lung capacity
UIP	usual interstitial pneumonia
